# Fast, streamlined fluorescence nanoscopy resolves rearrangements of SNARE and cargo proteins in platelets co-incubated with cancer cells

**DOI:** 10.1186/s12951-022-01502-w

**Published:** 2022-06-21

**Authors:** Jan Bergstrand, Xinyan Miao, Chinmaya Venugopal Srambickal, Gert Auer, Jerker Widengren

**Affiliations:** 1 Department of Applied Physics, Experimental Biomolecular Physics, Albanova Univ Center, Royal Institute of Technology (KTH), 106 91 Stockholm, Sweden; 2 Department of Oncology-Pathology, K7, Z1:00, Karolinska University Hospital, Karolinska Institutet, 171 76 Stockholm, Sweden

**Keywords:** STED, Super-resolution microscopy, Platelet, Cancer, Tumorigenesis, SNARE protein, Dictionary learning

## Abstract

**Background:**

Increasing evidence suggests that platelets play a central role in cancer progression, with altered storage and selective release from platelets of specific tumor-promoting proteins as a major mechanism. Fluorescence-based super-resolution microscopy (SRM) can resolve nanoscale spatial distribution patterns of such proteins, and how they are altered in platelets upon different activations. Analysing such alterations by SRM thus represents a promising, minimally invasive strategy for platelet-based diagnosis and monitoring of cancer progression. However, broader applicability beyond specialized research labs will require objective, more automated imaging procedures. Moreover, for statistically significant analyses many SRM platelet images are needed, of several different platelet proteins. Such proteins, showing alterations in their distributions upon cancer progression additionally need to be identified.

**Results:**

A fast, streamlined and objective procedure for SRM platelet image acquisition, analysis and classification was developed to overcome these limitations. By stimulated emission depletion SRM we imaged nanoscale patterns of six different platelet proteins; four different SNAREs (soluble N-ethylmaleimide factor attachment protein receptors) mediating protein secretion by membrane fusion of storage granules, and two angiogenesis regulating proteins, representing cargo proteins within these granules coupled to tumor progression. By a streamlined procedure, we recorded about 100 SRM images of platelets, for each of these six proteins, and for five different categories of platelets; incubated with cancer cells (MCF-7, MDA-MB-231, EFO-21), non-cancer cells (MCF-10A), or no cells at all. From these images, structural similarity and protein cluster parameters were determined, and probability functions of these parameters were generated for the different platelet categories. By comparing these probability functions between the categories, we could identify nanoscale alterations in the protein distributions, allowing us to classify the platelets into their correct categories, if they were co-incubated with cancer cells, non-cancer cells, or no cells at all.

**Conclusions:**

The fast, streamlined and objective acquisition and analysis procedure established in this work confirms the role of SNAREs and angiogenesis-regulating proteins in platelet-mediated cancer progression, provides additional fundamental knowledge on the interplay between tumor cells and platelets, and represent an important step towards using tumor-platelet interactions and redistribution of nanoscale protein patterns in platelets as a basis for cancer diagnostics.

**Supplementary Information:**

The online version contains supplementary material available at 10.1186/s12951-022-01502-w.

## Introduction

Experimental evidence increasingly highlights platelets as active players in all steps of tumorigenesis, including tumor growth, tumor cell extravasation and metastasis [1–4]. Platelets can adhere to circulating tumor cells (CTCs), to help CTCs evade immune surveillance, survive shear stress of blood flow, and promote their tethering and arrest to capillary blood vessel walls. By direct binding, or via secreted platelet-derived microparticles (PMP:s) and mediator molecules, activated platelets can induce tumor growth, epithelial-mesenchymal transition, and promote angiogenesis and tumor cell establishment at distant sites [1, 3, 5–7]. Vice versa, tumor cells can also activate platelets in multiple ways, resulting in so-called tumor-educated platelets (TEPs) [6, 7]. Release of cytokines from tumor cells can drive platelet overproduction by the megakaryocytes in the bone marrow [7, 8]. CTCs can bind directly to specific molecules on the platelet plasma membrane (PM), or activate platelets by extracellular release of bioactive compounds, such as thrombin, adenosine diphosphate (ADP) and thromboxane A2 (TXA2). Moreover, tumor-derived proteins or RNA can be sequestered by platelets by uptake of extracellular vesicles (EVs) from tumor cells [5, 6].

Altered contents of specific proteins in circulating platelets have been found both in mice bearing human malignant tumor xenografts [9, 10], and in patients with different metastatic diseases [11]. Such alterations may already be present in platelets when budded off from the megakaryocytes, or take place via RNA uptake from EVs, followed by altered protein expression by the platelets themselves [5, 6]. However, they have also been ascribed to activation-specific protein storage, release and uptake in the platelets [12–15]. While the mere content of specific proteins in the platelets can be found by several methods, including 2D-electrophoresis, mass spectrometry, fluorescence-based flow cytometry and confocal laser scanning microscopy (CLSM) [11, 12, 16], it is still an open question how platelets specifically can regulate uptake and release of certain proteins. The advent of fluorescence-based super-resolution microscopy (SRM) now opens for detailed characterization of spatial distribution patterns of specific proteins within platelets, which can lead to an increased understanding [17]. This can be important for the development of new treatment regimens interfering with tumor-platelet interplay and for allowing certain activation states of platelets to be identified for diagnostic purposes.

The alpha-granules are the largest (200–500 nm in diameter), most abundant secretory granules (50–80/cell) in platelets, containing a variety of proteins regulating angiogenesis, coagulation, cell proliferation, adhesion, and immune responses [6, 13, 14, 18]. From platelet releasate [16] and CLSM co-localization studies [12] it has been suggested that selective release of proteins is due to different sets of proteins in the individual alpha-granules, which then can be selectively released depending on stimulus. Differential packaging of proteins into alpha-granules has been reported to take place already upon platelet formation in the megakaryocytes [19]. However, higher resolution electron microscopy (EM) [20, 21] and fluorescence super-resolution microscopy (SRM) [22, 23] studies rather indicate that proteins are stored in clusters within individual alpha-granules, down to 50 nm in diameter, and with highly segregated protein cargo. Other studies suggest kinetic differences in the release as a mechanism for selectivity [14, 24, 25], and intra-granular protein cargo segregation with alternative routes to and fusion with the open canalicular system (OCS) and the PM [13].

The specific protein uptake and release mechanisms in platelets also include specific proteins mediating membrane fusion, so-called SNAREs (soluble N-ethylmaleimide factor attachment protein receptors). SNAREs are highly evolutionary conserved proteins, found in almost all living organisms, from yeast cells to humans. Depending on location they are classified as target and vesicle SNAREs (t/Q- and v/R-SNAREs), the latter category also known as vesicle-associated membrane proteins (VAMPs) [26]. For platelet secretion, in particular the v-SNAREs VAMP7 and VAMP8 [27], and the t-SNAREs SNAP23 [28] and Syntaxin-11 (STX11) [29] have been reported to play critical roles.

When activated, specific protein uptake and release mechanisms in platelets should lead to a redistribution of different proteins within the platelets, of cell adhesion proteins in the alpha-granule membranes and in the OCS and PM, of cargo proteins in the alpha granules, as well as of SNAREs required for platelet secretion. Fluorescence-based SRM techniques are well suited to identify and resolve such protein redistributions, and have emerged as promising tools to study tumor cell-platelet interactions in general [14, 17, 30]. In previous work [23, 31], we introduced fluorescence-based, stimulated emission depletion (STED) SRM to study distribution patterns of specific proteins in platelets upon distinct activations by well-known platelet activators (thrombin and ADP). Among the proteins studied [pro-angiogenic VEGF, anti-angiogenic PF-4 and Fibrinogen (Fg)], we found clear differences in sizes, numbers and spatial distributions of their regional clusters within the platelets following activation. No significant co-localization between the proteins was found, indicating that the proteins are stored differently in the platelets, and the rearrangements that took place in the platelets upon activation were found to be specific for a certain protein and activation, indicating different release and uptake mechanisms. More recently [32], we demonstrated that STED imaging can not only detect specific protein distribution patterns in platelets upon distinct activation by known agents, but can also detect specific patterns arising when different cancer cells are co-incubated with the platelets. Specifically, distinctive changes in the spatial distribution patterns were found for the cell adhesion protein p-selectin, a biomarker for platelet activation, which is often increased in cancer patients [33, 34]. The changes were to a significantly lesser extent, if at all, found in platelets incubated with normal cells. By developed image analyses of the p-selectin distribution patterns in the platelet STED images, it was then possible to classify platelets exposed to cancer cells, non-activated platelets, and platelets exposed to non-cancer cells or to soluble activators, in an objective manner. These platelet studies [23, 31, 32], indicate the potential of images resolving high-resolution spatial distribution patterns of biomolecules in cells as a source of diagnostic information. This particular information is not within reach by CLSM, EM and other techniques [17, 35].

In this work, we extend our STED SRM studies to additional proteins implicated in platelet secretion processes. We studied the spatial distribution patterns in platelets of v-SNAREs (VAMP7 and VAMP8) and t-SNAREs (SNAP23 and STX11), known to be central for platelet secretion [27–29], as well as patterns of the granule cargo proteins; vascular endothelial growth factor-A (VEGF) and thrombospondin-1 (TSP1), well known pro- [8] and anti-angiogenic [36] regulators, respectively. We investigated changes in the protein distribution patterns upon co-incubation of the platelets with cells from either a non-cancer (MCF-10A), an ovarian cancer (EFO-21), or from a breast cancer (MCF-7 and MDA-MB-231) cell line. For these proteins, and in contrast to our recent study of p-selectin [32], no distinct changes were observable in the STED SRM images by the naked eye. However, by a combination of analyses, including machine learning, structural similarity and cluster analyses, and by having a larger number of platelets included, classification of differently activated platelets is possible. These analyses, combined with a streamlined, higher throughput STED image acquisition, as established in this work, thus shows promise as a diagnostic tool and for fundamental platelet physiology studies. Specifically, the approach presented in this work is capable to resolve nanoscale distributions of SNARE proteins in platelets upon cancer cell incubation, confirming the role of these proteins in selective protein storage and release in platelets.

## Material and methods

### Platelet preparation

The preparation of platelets followed the same procedures as previously described [23, 31, 32]. In short, venous blood was drawn from healthy donors, having no medicine intake within two weeks before sampling, collected in 3.8% (g/ml) trisodium citrate, and centrifuged (150*g*, 20 min) at RT without break. The platelet rich plasma (PRP) fraction was then obtained by collecting the upper two thirds of the centrifuged blood. The platelet samples used in the classification were obtained from several (3–4) different donors.

Four different cell-lines, including two breast cancer cell lines (MCF-7, MDA-MB-231), one ovarian high-grade serous cancer cell-line (EFO-21), and one immortalized non-cancer cell line (MCF-10A), were used for co-culturing with platelets. 500 μl of the cell suspensions (3 × 10^5^ cells/ml) was seeded on poly-l-lysine-coated coverslips in a 24-well plate and incubated at 37 °C for 6 h. 2 × 10^7^ platelets in enriched PRP were then added to each well in 600 μl, together with serum-absent medium to avoid nonspecific activation of the platelets. Suspensions of the freely diffusing platelets were incubated with the cells for 2 h at 37 °C, and then fixed with 2% paraformaldehyde. The fixed platelets were spun down (1000*g*, 10 min, 22 °C), and then diluted with an equal amount of Tyrodes buffer.

The fixed platelet suspension was seeded on poly-l-lysine-coated coverslips in Nunc™ 4-Well Dishes, and the platelets were then allowed to adhere to the coverslip by gentle centrifugation. Resting platelet samples were also prepared as described above without any co-culturing.

### Immunostaining

The immunostaining essentially followed previously established protocols [23, 32]. Donkey anti-rabbit and goat anti-mouse antibodies (Sigma), conjugated with the fluorophores Alexa 594 (Thermo Fisher Scientific) and ATTO 647 N (Atto-Tec GmbH, Siegen, Germany), respectively, were used as secondary antibodies. Mouse monoclonal antibodies (Santa Cruz Biotechnology, Heidelberg, Germany) were used for targeting human STX11, TSP1, VEGF and SNAP23. Rabbit polyclonal antibodies (Abcam) were used to target VAMP7, and rabbit monoclonal ones (Santa Cruz Biotechnology, Heidelberg, Germany) to target VAMP8. DPBS was used as buffer. Fixed platelets were permeabilized with Triton X-100 (0.5%, 10 min), blocked for nonspecific binding with bovine serum albumin (1% w/v, Sigma, Sweden) for 1 h at RT, then washed three times with DPBS, and incubated overnight (4 °C, moist atmosphere) with primary antibodies (2 μg/mL). The samples were then washed three times with DPBS, incubated with either of the secondary antibodies (3 μg/mL) for 1 h in RT, and then washed three times before being mounted onto microslides with Mowiol-Dabco mounting medium (Sigma, Sweden). Typically, for the different combinations of protein stainings and co-culturing conditions only one stained sample slide was prepared per biological sample to keep down the total number of stainings. However, for VAMP8, duplicate stainings made for different co-culturing conditions allowed controls of reproducibility in the classification between technical replicate samples.

### Stimulated emission depletion (STED) imaging

STED imaging was performed on a modified 2-colour STED instrument (Abberior Instruments GmbH, Göttingen, Germany), built on a stand from Olympus (IX83), see [32] for further details. In short, it operates with pulsed excitation (at 594 nm and 638 nm), and a pulsed STED beam at 775 nm. Image acquisition, including laser timing/triggering and detector gating, is controlled via an FPGA-card and by the Imspector software (Abberior Instruments GmbH). To reduce noise and increase contrast in the STED images, the freely available “Iterative Deconvolve 3D”-class in ImageJ was used as a plugin with a moderate number of maximum 10 iterations per image. With this number of iterations, artifacts in the images due to deconvolution were found to be small. No Wiener-filtering was used (option turned off), while an option to avoid ringing effects was turned on in the deconvolution algorithm. A low pass (gaussian) filter set to one pixel was included to reduce the photon (“salt & pepper”) noise in the images. A point spread function (PSF) of 40 nm in the radial direction was considered for the deconvolution, corresponding to the microscope PSF at the measurement conditions. In the axial dimension, the extension of the PSF was diffraction-limited, and estimated to 600 nm. Effective imaging resolutions (taking e.g., the size of labeling antibodies into account) were determined using Fourier ring correlation (FRC) [37]. The (radial) resolutions were determined to 43 nm and 48 nm, before and after deconvolution, respectively. (See Additional file 1: Figs. S1 and S2). There was thus no significant loss of resolution as a result of the deconvolution. Moreover, given that the deconvolution algorithm was applied the same to all raw data images, and what we consider in the classification in the end are differences between the samples, we expect any changes in the images introduced by the deconvolution to have negligible effects on the classification.

### Streamlined STED imaging

The microscope is controlled by the Imspector software (Abberior Instruments GmbH). Via a Python interface to Imspector (SpecPy), we wrote a custom script to control every part of the microscope in a more automated way, allowing us to image a large number of platelets within an area predefined by the user. This customized algorithm makes it possible to record STED images of a large quantity of platelets in a significantly shorter time, compared to recording every platelet one by one in a manual manner. It essentially works in three steps (see also main text for further details):

1. The user manually selects an area (typically 50 × 50 μm) in the sample and a coarse confocal image of the platelets is recorded. The algorithm then identifies each platelet within this image by identifying pixels with values above a predefined threshold. The coordinates for the center of intensity of each platelet is stored in an array, and a box is drawn around each platelet, whereby the user can manually inspect whether the algorithm successfully identified the platelets or not. If platelets are unsuccessfully identified, the user can choose to terminate the algorithm and choose another area, or re-define intensity thresholds for the identification step above.
2. The coordinates for each identified platelet are sent to the scanner of the microscope, and the optimal optical focus for each of the individual platelets is refined by an intermediate automatic focusing algorithm. In this focusing algorithm the microscope performs 10 fast xy-scans of the platelet (with a large pixel size and short dwell time so that scanning time is less than 0.5 s per frame), producing images at different focal planes with 200 nm space in the z-axis between them. Given an axial, diffraction-limited resolution of 600 nm, and since we are imaging platelets with discoidal shapes of low thickness, a step size of 200 nm should be sufficient to properly position the sample in the axial direction. In our case, no further improvements in the autofocus procedure are thus to be expected by using shorter step-sizes. Moreover, with shorter step-sizes, additional axial steps (and imaging rounds) typically follow before reaching a proper axial position, which adds to the photobleaching [38]. For each image recorded, an image of the intensity gradient to its upper and lower neighbor images is calculated and the focal plane of the image with the largest sum of its gradient image is defined to be the optimal focus. Based on extensive testing and comparing with manually setting the focus, this automatic procedure produces a focus setting which is equivalent to manually setting the focus, with a focus setting within ± 200 nm of a manually set focus.
3. With the optimal focus for an individual platelet defined in step 2, the algorithm puts all the parameters of the microscope in a state such that a high quality STED image is automatically recorded for this specific individual platelet. This is repeated for each identified platelet within the manually chosen large area in step 1 until all of the platelets have been recorded. All platelet images are then saved in a predefined file format, along with any optionally user defined metadata. Once the algorithm stops it can be repeated from step 1 by the user selecting a new area within the sample.

### Structural similarity (SSIM) analysis

SSIM analyses were performed on platelet images as previously described [32], implementing the Scikit package [39], a free Python package for utilizing different kinds of machine learning tools. First, computer-simulated platelet images were generated to have cluster-like structures randomly distributed within a defined area resembling the area of a platelet (see Additional file 1 for additional details). A dictionary with 9 × 9 image elements of 30 × 30 pixels each was then computationally generated from these simulated images as a training set. While training the dictionary, we enforced sparsity, i.e., that images in the training data are expressible as a linear combination of the elements in the dictionary using the least possible number of elements. While an infinite number of elements in the library in principle makes it possible to reconstruct any image as a linear combination of these elements, we in fact used the inability of a limited, sparse number of elements to re-create the images, and how the extent of this inability varied between different sample categories, as a distinguishing feature. The dictionary was next used to reconstruct images from the different sets of the experimental images. Each reconstructed image was then compared to its original image using the Structural Similarity (SSIM) norm [40], which returns a number between 0 and 1 depending on how good the reconstruction is (and where SSIM = 1 represents a perfect reconstruction). The SSIM values for the experimental STED images, i.e., how well they could be reconstructed from the best possible linear combination of dictionary elements, were then used as a basis for classification. See [32] for further details.

### Cluster analysis

Dual colour STED images of multiple proteins pairs were imaged, generating pairs of spectral channel images of the specific proteins. Following iterative deconvolution, as described above, the images corresponding to individual proteins were separated and further analyses were performed using a custom written Python program. Each image was subject to automatic Otsu thresholding (from the Scikit-image package [39]) to obtain a binary image of clusters before finding the intensity independent center of mass of the protein clusters. A circular area with a radius of 1.6 μm from the center of mass, large enough to cover the clusters within all platelets, was used for the calculation of total number of clusters, *N*_*C*_, per platelet. *N*_*C*_ for each platelet image was determined with the feature labelling function available in the SciPy image processing toolbox, in which pixels which have a square or a diagonal connectivity are grouped into clusters and counted. The mean cluster area, *A*_*C*_, from each image was calculated from dividing the total area of the clusters within the platelet by the total number of clusters.

### Radial analysis

To determine the protein radial distribution, each image was divided into 10 concentric zones of equal width (250 nm) around the center-of-mass of the protein clusters. The protein clusters within each zone were then counted to obtain the radial cluster distribution, $N\left(r\right)$. The image processing, including thresholding center of mass calculation and cluster counting were performed as described above for the cluster analysis.

Furthermore, $N\left(r\right)$ was normalized such that the sum of clusters over all zones in $N\left(r\right)$ equals one, i.e., $\sum_{r}N\left(r\right)=1$. For the normalized radial distribution, $\overline{N}\left(r\right)$, we calculated the first and second order moment $m1$ and $m2$. $m1$ corresponds to the average distance of clusters from the center of mass of the particular platelet under consideration, and $m2$ relates to the variance of the distance of the same radial distribution of protein clusters. $m1$ and $m2$ are given by:

1 $$m1=\sum_{i}{r}_{i}N\left({r}_{i}\right)$$

2 $$m2=\sum_{i}{\left({r}_{i}\right)}^{2}N\left({r}_{i}\right)$$

Here *r*_*i*_ is the outer radius of the concentric zone *i* from the center of mass of the platelet and $N\left({r}_{i}\right)$ is the number of protein clusters within the i:th concentric zone.

### Probability functions generated via Kernel density estimates (KDEs)

The KDE plots were generated using Scikit Learn python package [39] with a gaussian kernel and the free parameter ‘bandwidth’ for individual datasets were determined in an objective manner using the grid search cross validation function GridSearchCV from Scikit Learn. All data points above a five-sigma cut-off were removed before finding the optimal bandwidth for parameters which had very large outliers (e.g., in the mean cluster area). Once the kernel density was estimated, a suitable range of parameter values were selected (avoiding the asymptotic tails of the density function) and binned to obtain a normalized probability function for each dataset.

## Results

### Platelet image acquisition

**Fig. 1:**
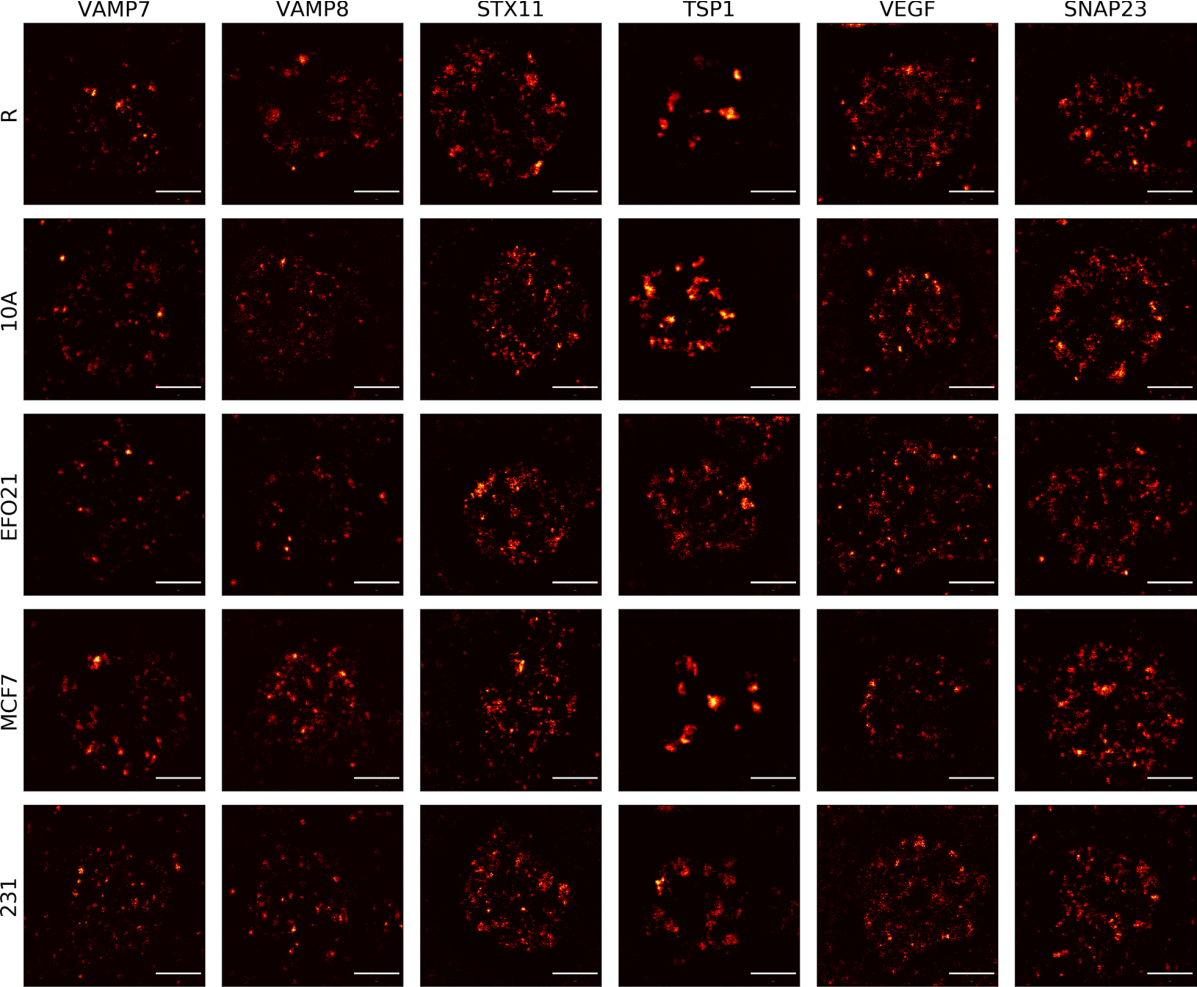
Representative STED images of platelets with different protein labels and subject to different co-culturing conditions. Platelets with different protein labels are imaged either under resting (R) condition, after co-culturing with cells from a non-tumor cell line MCF-10A (10A), or from different tumor cell lines MDA-MB-231 (231), EFO-21 (EFO21) and MCF-7 (MCF7). Scale bar: 1 µm

Distribution patterns within platelets were imaged by STED microscopy for 6 different proteins, all reported to play a role in tumor-mediated protein storage and release in platelets [8, 19, 27–29, 36]: VAMP7 (v-SNARE), VAMP8 (v-SNARE), VEGF (pro-angiogenic), TSP1 (anti-angiogenic), STX11 (t-SNARE) and SNAP23 (t-SNARE). The distribution patterns for these proteins were studied under resting and four different (co-)culturing conditions (abbreviation in parentheses): resting (R), co-culturing with non-cancer cell-line MCF-10A (10A), with cancer cell line MCF-7 (MCF7), with EFO-21 (EFO21) or with MDA-MB-231 (231). The R and 10A platelets were used as controls in order to distinguish between tumor exposed platelets and platelets not exposed to tumor cells. Typical STED images of platelets labeled for the different proteins and subject to the different conditions are shown in Fig. 1. In total, we recorded more than 3000 platelet images, roughly distributed equally over the different proteins. We thus acquired about 500 experimental images for each of the proteins and about 100 platelet images for each combination of protein (VAMP7, VAMP8, STX11, TSP1, VEGF, SNAP23) and co-culturing condition (R, 10A, MCF7, EFO21, 231). We estimate that our streamlined image acquisition algorithm (described in Methods section) can record individual STED images of platelets at least twice as fast as by manual recording of the platelet images. This can likely be faster by further refinement of the algorithm in the future. This algorithm thus makes it possible to get larger data sets in less time and with less effort, providing better statistics in the subsequent analyses, to distinguish between tumor cell co-cultured platelets (MCF7, EFO21, 231) from resting platelets (R) and non-tumor cell co-cultured platelets (10A).

### Analyses of nanoscale protein distribution features in the platelet images

**Fig. 2:**
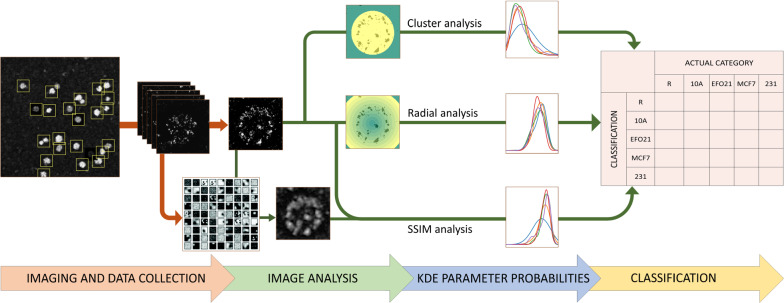
Overall workflow for the acquisition of platelet images, analysis of nanoscale protein distribution patterns and classification based on probabilities for image parameters measured. See text for more details

By visual inspection of the recorded STED images (Fig. 1), no evident differences in the distribution patterns of the different proteins could be observed in platelets subject to different co-culturing conditions. To distinguish the different platelet categories from each other, we established an objective, streamlined algorithm also for the analyses, following an overall workflow, as visualized in Fig. 2.

The recorded images were first processed in ImageJ by a deconvolution algorithm. Care was taken so that the deconvolution did not introduce significant artifacts (see Methods sections for details). Second, all sets of images (6 different proteins, 5 different conditions) were analyzed based on three different analysing strategies (Fig. 2): First, a machine learning approach based on dictionary learning and SSIM analyses was applied, then, protein cluster analyses, analyzing the overall number (*N*_*C*_) and mean area (*A*_*C*_) of the protein clusters within each platelet. Finally, an approach was applied analyzing how the clusters were distributed radially, within radial sections from the center of mass of each of the platelets.

In the dictionary learning process a dictionary of image elements is built (trained) from a larger number of example images (training data). While we recorded more than 3000 images in total, about 100 platelet images per protein and co-culturing condition is not sufficient to make up such training data sets, for each combination of imaged protein and co-culturing condition (R, 10A, MCF7, EFO21, 231). We therefore generated 20,000 simulated images, with randomly distributed protein clusters within a defined area resembling the area of a platelet as a basis for the dictionary training, for all different combinations of protein and co-culturing condition.

Linear combinations of the elements from the resulting trained dictionary (with 9 × 9 elements, 30 × 30 pixels each) was then used to reconstruct an image from each of the experimental platelet images. An SSIM norm was then assigned to every experimental platelet image for every protein and (co-)culturing condition, yielding a number between 0 and 1, depending on the degree of similarity between each reconstructed image and the original experimental image (see Methods and [32]).

Similarly, protein clusters were identified in the platelet images, whereby each platelet was assigned with its protein cluster number (*N*_*C*_) and the mean area of its clusters (*A*_*C*_), as described in the Methods section. Finally, the normalized radial cluster distribution, $\overline{N}\left(r\right)$ was determined for each imaged platelet, and then used to calculate the first and second radial moment, *m1* and *m2* respectively (see Methods).

### Probability distributions of platelet parameters via Kernel Density Estimates

**Fig. 3:**
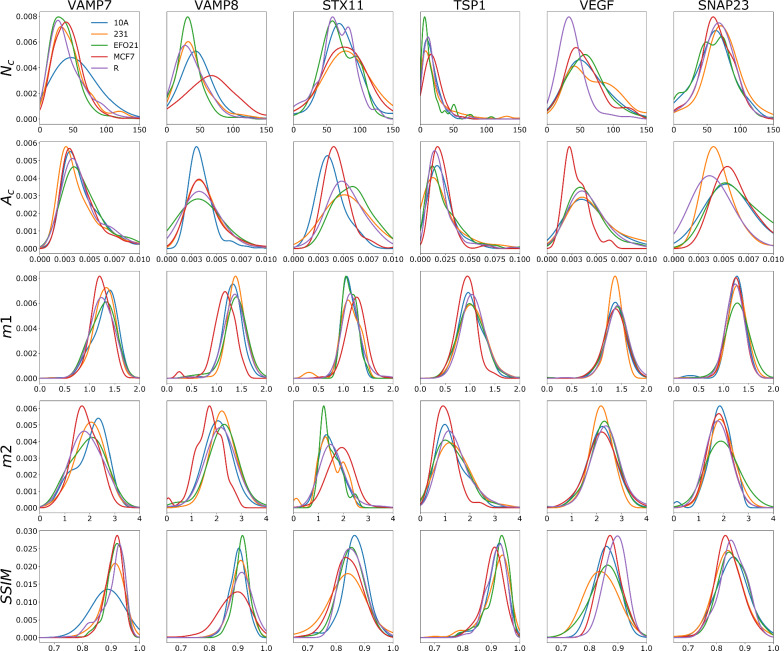
Normalized KDE probability functions for the different platelet image parameters, for each protein studied and under each of the different co-culturing conditions. The parameters are total number of clusters (*Nc*), mean area of cluster (*Ac*), first (*m1*) and second order moment (*m2*) of the radial distribution of clusters within a platelet, and the Structure Similarity Index Measure (*SSIM*), calculated by comparing the platelet image with its corresponding reconstructed image using dictionary learning (See text for more details). The x axis for each plot correspond to the respective parameter value (units: a.u for *Nc* and *SSIM*, µm^2^ for *Ac* and *m2*, µm for *m1*. The y axes show the normalized probability (a.u)

From the platelet image analyses above, five parameters (*N*_*C*_, *A*_*C*_, m1, m2 and *SSIM*-norm) were assigned to all the individual platelets imaged. From the distribution of each of these parameter values, for platelets within each of the 30 different categories (the 6 × 5 combinations of protein imaged and (co-)culturing condition) corresponding probability functions were then obtained by Kernel Density Estimation (KDE), see Methods for further details. The complete normalized probability functions for all combinations of the 5 platelet categories, 6 proteins and 5 platelet image parameters are displayed in Fig. 3. From Fig. 3, it can be noted that despite there being no significant differences between different platelet images (Fig. 1) upon visual inspection, the probability functions of Fig. 3 show distinct differences in the distributions between different co-culturing conditions for many of the proteins and calculated parameters. For example, *N*_*C*_ of VEGF in the resting platelets (R) are much lower than in platelets co-cultured with cells from any of the cell-lines used. For VAMP7, platelets subject to the 10A co-culturing condition have generally lower *SSIM* values than platelets in the other categories. For VAMP8, the *SSIM* probability function for platelets co-cultured with MCF7 tumor cells shows the same trend. It can also be noted that even for parameter probability functions which have similar average values, their standard deviations and shapes can be identifiably different, e.g. *A*_*C*_ for VAMP8 and *m2* for STX11.

Overall, the observed differences in the probability functions between platelets subject to different (co-)culturing conditions indicate that several of the proteins in the platelets are indeed redistributed upon cellular co-incubation, and that this redistribution is different depending on what category of cells the platelets have been co-cultured with. Specifically, since some of the proteins in platelets exposed to tumor cells seem to change their distribution patterns differently, compared to platelets exposed to non-tumor cells, we next designed a custom classification pipeline to classify sets of platelets into different (co-)culturing categories based on these differences.

### Classification of platelets based on STED images of the individual different proteins

**Fig. 4:**
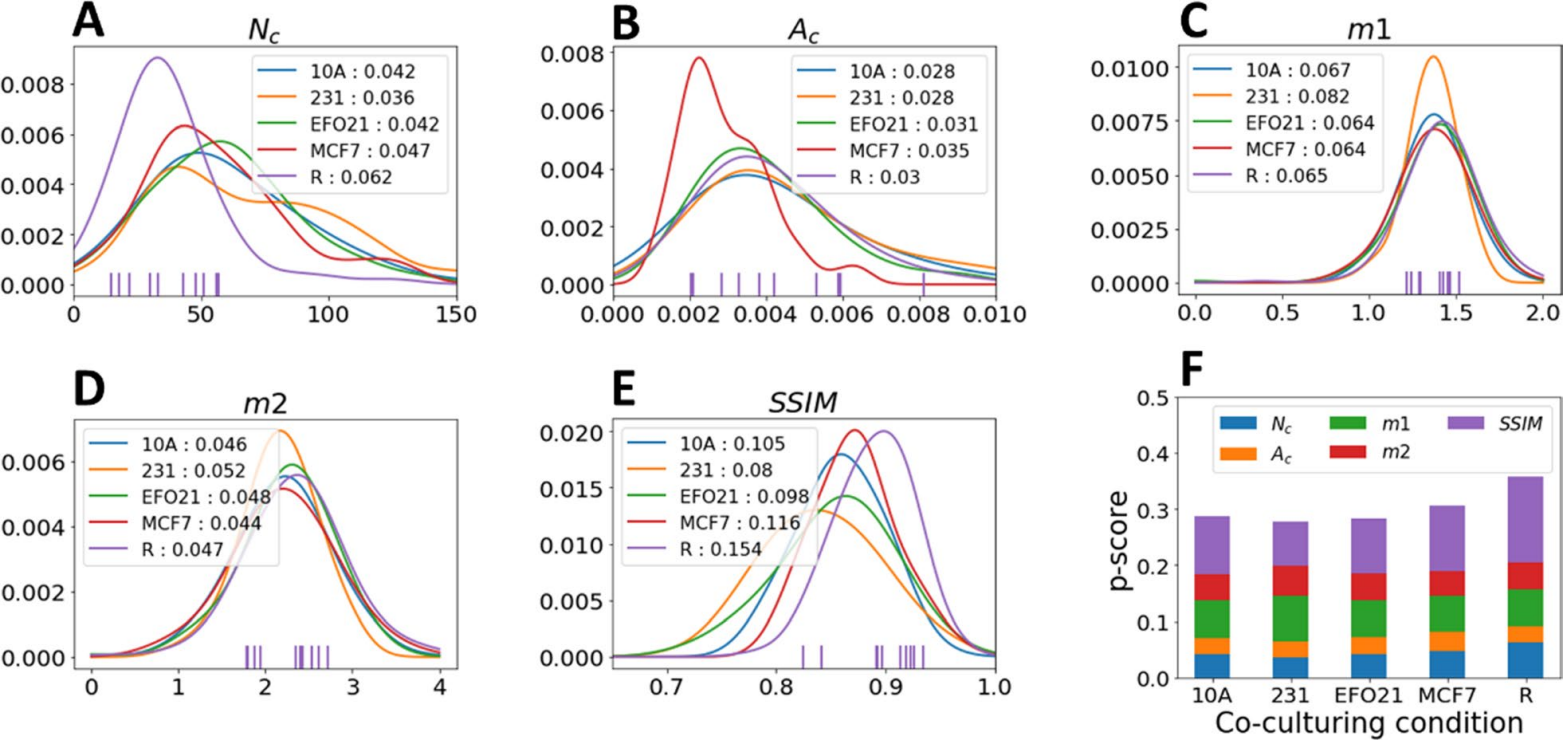
Example of *p-score* calculation (In this case based on images of platelets with VEGF staining). A set of 10 platelets from a certain (co-)culturing category are randomly selected (in this example from resting (R) platelets). For each of the 5 imaging parameters, the values determined for the 10 platelets are compared against the corresponding probability curves, in this example for VEGF, and for all the different platelet categories (**A**–**E**). The probabilities of the 10 platelets to belong to a certain category are then added for each parameter (**A**–**E** insets). **F** The combined *p-score* calculated for each co-culturing condition is obtained by adding the sum probabilities for all parameters for the given culturing category. The set of 10 platelets are finally classified into the category with the highest combined *p-score*, in this example into R

To test to what extent differences in the probability functions of Fig. 3 can be used for classification of platelets into the different categories, we devised a procedure based on classifying iteratively 10 platelets at a time, as exemplified in Fig. 4. The parameter sets (*N*_*C*_, *A*_*C*_, *m1*, *m2*, *SSIM*) from 10 platelets of a given (co-)culturing category were selected in a randomized manner among all the imaged platelets in that category, and for one of the six proteins at a time. For the 10 selected platelets, the set of 10 values for each of the parameters were then compared against the KDE probability curves in Fig. 3, for the corresponding parameter and protein, and for each of the different platelet categories (exemplified in Fig. 4A–E). Thereby, the probability to actually get the sets of 10 values for each of the five parameters was compared between the different platelet categories. (In the example of Fig. 4A–E, these probabilities are shown in the corresponding insets, for each of the five parameters). Next, the sums of these probabilities for the five parameters (*N*_*C*_, *A*_*C*_, *m1*, *m2*, *SSIM*) were then in turn added into a probability score *p* for each of the co-culturing categories (Fig. 4F). Finally, the set of 10 test platelets was then classified into the category with the highest *p*-score [In the example of Fig. 4, 10 resting platelets images with VEGF staining were selected and classified into the resting (R) category, since the *p*-score was the highest for R (0.36)].

**Fig. 5:**
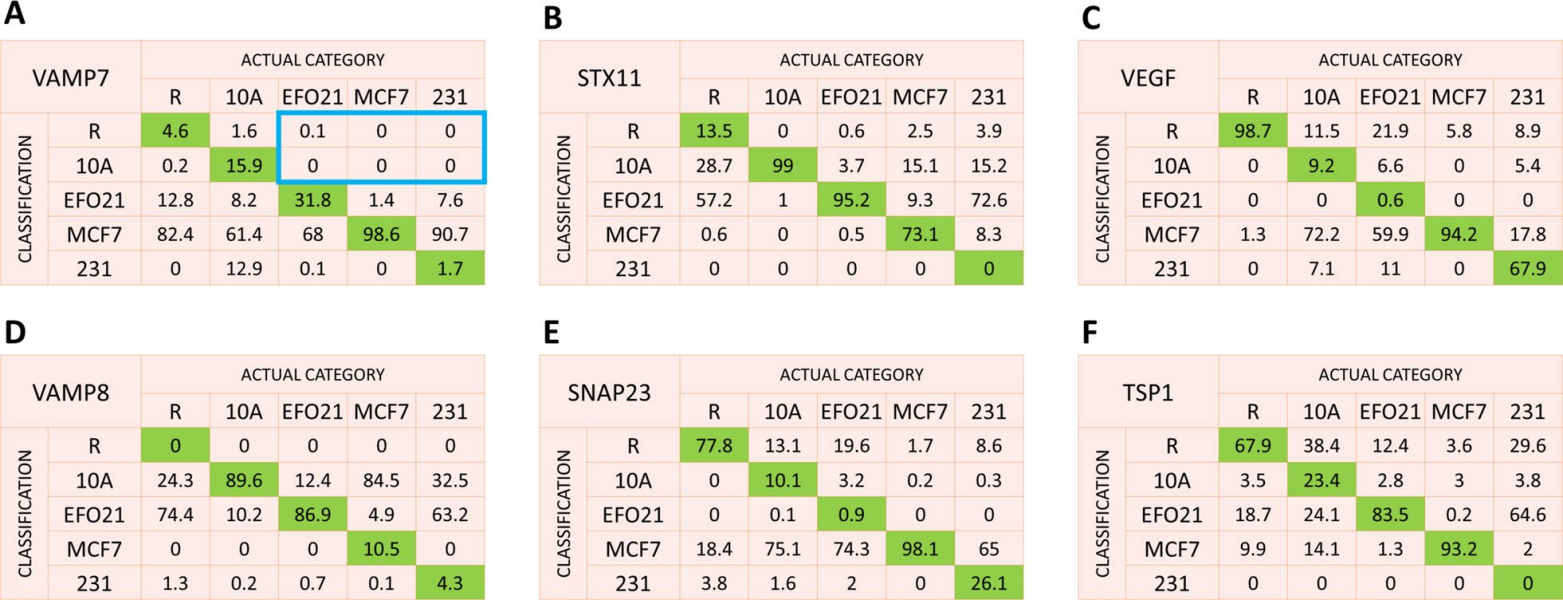
Classification probability matrices (in %) for individual proteins to classify a set of ten platelets of an actual category (columns) into a certain category (lines). The matrices are generated by randomly selecting 1000 sets of ten platelets from a known category and classifying them into one of the five categories. Probabilities enclosed in blue represent false negative classifications of tumor educated platelets

To build classification probability statistics we used a boot-strapping approach, in which 10 platelet images were randomly selected and classified into the category reaching the highest *p*-score, as described above, and where this platelet selection and classification was repeated 1000 times among the platelet images for each of the 5 co-culturing categories and for each of the 6 imaged proteins. Figure 5 shows the resulting classification probability matrices from this approach for the individual proteins. The diagonal values on the matrices represent the probabilities that ten platelets of a certain category and imaged with respect to a given protein are classified correctly into their true category. It can be noted that in the classification probability matrices of the different proteins in Fig. 5, the classification probabilities are quite high in some cases. However, there are also some high off-diagonal elements in the matrices representing false classifications. For example, from images of VAMP7, platelets co-cultured with MCF7 tumor cells can be correctly classified with very high probability (98.6%). However, such high true positive probabilities were not achieved for any of the other platelet categories with this protein. Moreover, with VAMP7 the probabilities to falsely classify the other categories to be MCF7 are also very high, such that the significance in correctly classifying a set of platelets with unknown activation to MCF7 using VAMP7 would be quite low. In Fig. 5, similar high true positive probabilities are seen also for resting (R) and 10A co-cultured platelets (10A) based on the VEGF and STX11 proteins, respectively. In contrast to VAMP7 and MCF7 however, the false positive probabilities are in these cases much lower, which improves their classification strengths.

As illustrated by the above examples, and as seen more generally in the matrices of Fig. 5, none of the proteins can provide high true positive classification probability for all the co-culturing categories. Rather, their classification strengths complement each other.

### Classification of platelets based on a combination of STED images of different proteins

To further refine and improve the classification of the different platelet categories, we next took advantage of these complementary classification strengths of the different proteins. For each of the 6 different proteins 10 platelet images were randomly selected from a given platelet category, $m$, (i.e., 60 platelet images in total) and each protein subset was classified into a category, $k$, as described above. With 5 different categories, both *m* and *k* take integer values between 1 and 5 (R = 1, 10A = 2, MCF7 = 3, 231 = 4, EFO21 = 5). Thereby, we generated a vector with 6 different classifications, $\overline{k }=({k}_{1},{ k}_{2}, { k}_{3}, { k}_{4}, { k}_{5},{ k}_{6})$, one from each of the proteins. From $\overline{k }$, and using the classification probability matrices for each protein in Fig. 5, we then defined a combined probability, ${\Psi }_{N}\left(m|\overline{k }\right)$, following a similar approach as previously used for single-molecule classification, [41] and where ${\Psi }_{N}\left(m|\overline{k }\right)$ expresses the probability that a set of 60 platelets from category *m* displays a certain $\overline{k }$, compared to the probability to obtain this $\overline{k }$ from any of the *M* categories:

3 $${\Psi }_{N}\left(m|\overline{k }\right)=\frac{\prod_{prot=1}^{N}{P}_{{k}_{prot},m}^{prot}}{\sum_{m=1}^{M}\prod_{prot=1}^{N}{P}_{{k}_{prot},m}^{prot}}$$

Here, ${P}_{{k}_{prot},m}^{prot}$, are the elements in the matrices of Fig. 5, describing the probability that a set of 10 platelets from a known category, *m*, and imaged with respect to a certain protein, *prot*, is classified as category ${k}_{prot}$, where ${k}_{prot}$ (*prot* = 1…*N*) are the elements in $\overline{k }$. *N* is the number of different imaged proteins used in the classification and *M* is the number of different platelet categories.

Similar to the classifications based on images of single proteins above (Fig. 4), we then used a boot-strapping approach to build classification probability statistics. For each of the *6* different proteins 10 platelet images were randomly selected from a given platelet category and $\overline{k }$ was determined. ${\Psi }_{N}\left(m|\overline{k }\right)$ was then calculated for all *m*, and the set of the *60* selected platelet images was thereafter classified into the category which yielded the highest ${\Psi }_{N}\left(m|\overline{k }\right)$.

**Fig. 6:**
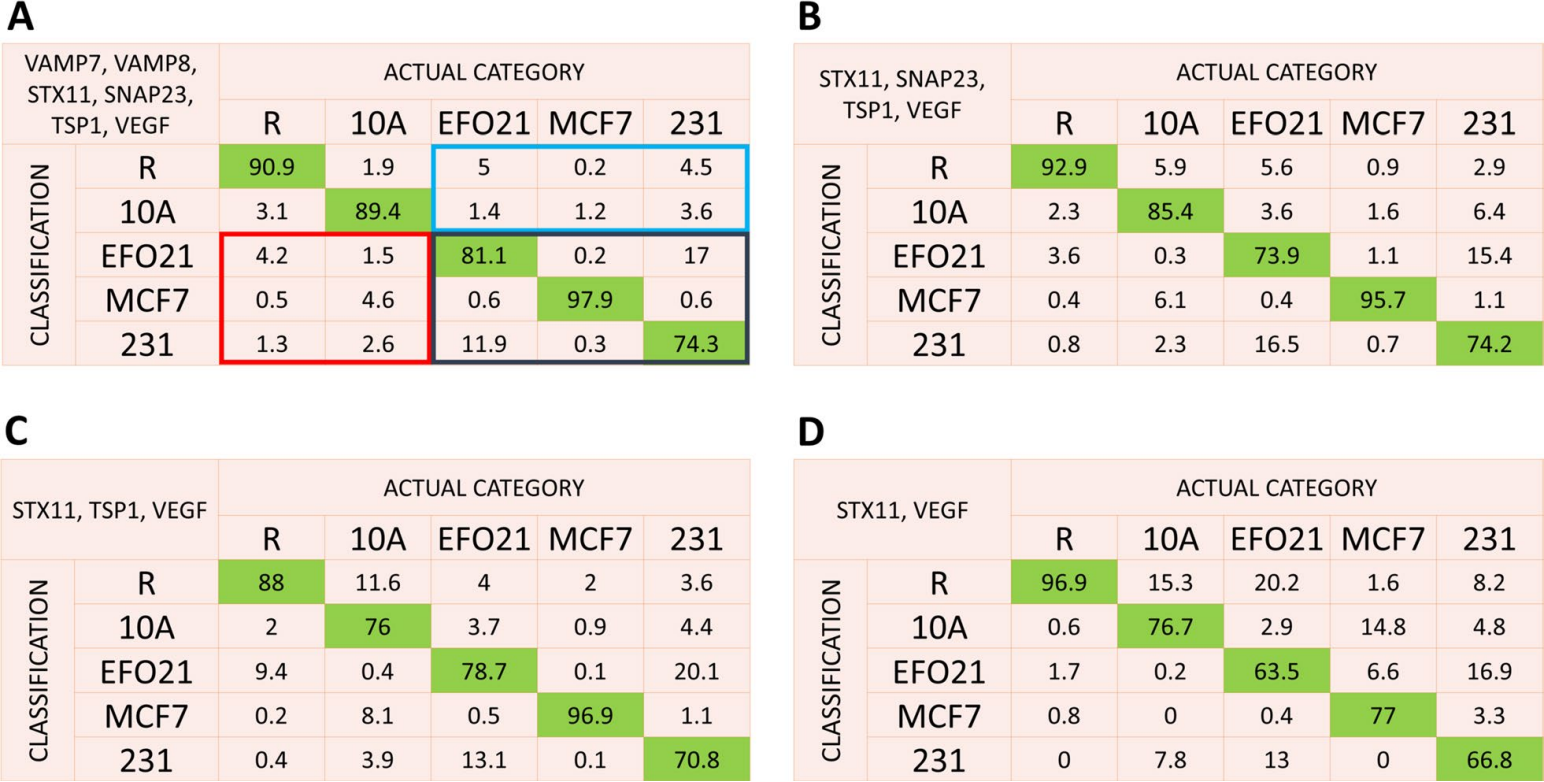
Classification matrices generated by combining the complementary classification strengths of different proteins. All possible combinations of 1 to 6 proteins were used for classification. The classification matrix for the best combination of **A** 6 proteins, **B** 4 proteins, **C** 3 proteins, and **D** 2 proteins. In **A** the probabilities enclosed in blue represent false negative classifications of tumor educated platelets (TEPs). Summing the probabilities enclosed in blue together, the average probability for false negative classification of TEPs, defined as the average probability to classify a platelet of category EFO21, MCF7 or 231 into the categories R or 10A is less than 6%. Similarly, the probabilities enclosed in red represent false positive classifications (with a probability < 8% when summed together), and those in black true positive classifications of TEPs (> 90% when summed together)

Classification probabilities were then calculated by randomly selecting and classifying sets of 60 platelet images (10 images from each of the six different proteins) recorded from platelets of known activation, and then repeating this procedure 1000 times. The resulting classification probability matrix, using images from all 6 proteins (N = 6) is shown in Fig. 6A. Indeed, the matrix of Fig. 6A is now overall strongly dominated by the diagonal elements, representing the probabilities for correct classifications. The two only larger (> 10%) off-diagonal elements represent probabilities to misclassify platelets co-cultured with different tumor cells (231 as EFO21, and vice versa). Considering the ability to classify if platelets have been co-cultured with tumor cells (EFO21, MCF7, 231) or not (R, 10A), it can be noted that the true classification probability exceeds 90%, and the probabilities for false positive and false negative classification are both lower than 8% (Fig. 6A).

Next, we considered which of the protein images are critical for the classification, and to what extent the number of different proteins studied, *N*, can be reduced. Following the procedure above, ${\Psi }_{N}\left(m|\overline{k }\right)$ was calculated from sets of *N*x10 platelets, from all combinations of the *N* proteins, and with *N* varied from 5 down to 2. Random boot-strapped selection was repeated 1000 times, and each selected set of *N*x10 platelet images was classified into the category yielding the highest ${\Psi }_{N}\left(m|\overline{k }\right)$, as described above. Classification probability matrices for all combinations of proteins are given in Additional file 1: Figs. S1–S6. Figure 6B–D show the resulting classification probability matrices, for the combinations of 4, 3 and 2 proteins yielding the highest diagonal elements. It can be noted that classification based on 4 proteins, not including VAMP7 and VAMP8, did not significantly reduce the diagonal elements, and also by use of two proteins only (STX11, VEGF) it is possible to generate a probability matrix with strongly dominating diagonal elements. The classification strength of these two proteins indicate that they are implicated in cell-platelet interactions, but also that the interactions are different for non-cancer and cancer cells.

## Discussion

In this work, we studied the nanoscale distribution patterns of 6 different platelet proteins regulating angiogenesis and vesicle fusion, implicated in tumor-educated platelet (TEP) formation. We show that nanoscale distribution patterns of these proteins in platelets, as imaged by STED SRM, can be used as a basis for classification of platelets, to distinguish between platelets co-cultured with cancer cells, non-cancer cell, or no cells at all. For the proteins studied, differences in their distribution patterns, on which the classification relies, can only to a very limited extent be seen by visual inspection of the STED images. However, we could devise a streamlined, objective procedure allowing classification of the different platelet categories with high fidelity. The effective resolution obviously sets a limit for the classification, in the sense that only features manifested on spatial scales longer than the resolution can be fully captured. Among the platelet imaging parameters used in the classification (*N*_c_, *A*_c_, *m*1, *m*2, and *SSIM*), *A*_c_ (average cluster area) is maybe the parameter most directly coupled to the resolution. Generally, the *A*_c_ values were found to be larger than the resolution limit, as measured by FRC, and differences in *A*_c_ values between the different sample categories were found on length scales beyond the effective resolution. With an effective resolution of 47 nm, *A*_c_ values below 0,002µm^2^ are not resolved. This would correspond to up to 20% of the range of the KDE plots (2% for TSP1). Extensions of the KDE probability functions into smaller areas are however generated as a result of the distribution of *A*_c_ values, beyond 0.002 µm^2^. In contrast, we see e.g., in Fig. 3 that for most proteins (except TSP1), their distributions of *A*_c_ (average cluster size) are within a range which cannot be resolved by diffraction-limited confocal imaging (resolution ~ 286 nm, as determined by FRC, see Additional file 1: Fig. S1). The presented classification thus requires the use of SRM to resolve the subtle differences in the protein distribution patterns between the different platelet categories. It also benefits from the higher throughput STED image acquisition and analyses introduced in this work, improving the statistical prerequisites for the classification. While the highest probabilities for correct classifications were obtained when STED images of all 6 proteins were included, comparable probabilities could also be obtained for a lower number of proteins. Among the individual proteins, STX11 (t-SNARE), VEGF (pro-angiogenic) and TSP1 (anti-angiogenic) showed the highest classification strengths (Fig. 5B, C and F), but not for all platelet categories. However, the distribution features of these proteins are complementary, in the sense that they display high classification strengths for different co-culturing conditions. High fidelity classifications based on these three proteins is thus also feasible (Fig. 6C), and even the use of only two of the proteins (STX11, VEGF) yields high incidences of correct classifications (Fig. 6D). The fact that STED images of these three proteins, individually or in combination, resulted in the highest classification strengths provide additional evidence that they are altered and play a role in TEP formation. On the other hand, while VAMP7 and VAMP8 can be excluded from the classification with relatively minor consequences, the classification matrix for VAMP7 (Fig. 5A) shows the overall lowest false negative classification probabilities, when discriminating between platelets co-incubated with cancer cells, with non-cancer cells, and with no cells at all. This can be taken as an indication that also VAMP7 is involved in TEP formation, although its spatial rearrangements upon the different co-incubations were not needed for the classification procedure devised in this work.

To address the possibility that observed feature differences would be due to experimental variability, we also compared classifications based on sets of images acquired from blood samples of different donors (biological replicates), as well as duplicate stainings from one and the same blood sample/donor (technical replicates). For at least one of the proteins, VAMP8, we could with certainty say that sets of images were recorded in different sessions, from separately stained slides, but from the same blood sample, and co-cultured in the same session. The outcome of the single protein classifications based on these different technical replicates are shown in Additional file 1: Fig. S9. Comparing the classification probabilities between the technical replicates, there is good reproducibility, especially it is very high for conditions where the classification strength of VAMP8 was found to be strong (10A and EFO21). Likewise, although we cannot compare the classification based on biological replicates for all proteins at the same time, we could compare sets of images of platelets from different samples/donors, stained for the same specific protein, and co-cultured in the same way. We then find reasonable reproducibility in the classification, between the identified biological replicates (particularly in the classification of platelets co-cultured with or without cancer cells), and in relation to the total classification outcome, based on the total group of platelets within each co-culturing category (Fig. 5). The outcome of the classification procedure for different proteins, on platelets subject to different co-culturing conditions, and compared between different biological replicates, are shown in Additional file 1: Fig. S10.

In the classification procedure, we introduced KDE-based probability distributions, and compared parameter values from several (here 10) platelet images to these probability distributions (Fig. 4A–E). While probability densities can be obtained by plotting histograms, they heavily depend on binning size and bin edges, which are user defined and arbitrary. This can also result in zero probability bins even though the underlying variable is continuous and smoothly varying. However, this is not a problem in KDE plots, which are obtained by summing over a kernel function placed at every data point. The only free parameter is the bandwidth of the kernel function, which can be assigned in an automated manner by cross-validation methods. The resulting KDE probability densities are smoother and more representative for the underlying actual probability distributions. Thereby, they can circumvent above mentioned problems associated with the use of plain probability histograms. In this way, we could accommodate distribution features of the parameter values into our classification, related to shape, spread and specific displacements of peak probabilities of the distributions for the different platelet categories. This has some relevance in a future scenario, where one may wish to classify clinical, and likely more heterogeneous, platelet samples, where sample heterogeneities can be part of the distinguishing features. Our approach, and the way features may vary within an ensemble of platelets, as much as the features within the individual platelets, may thus be used to tell two different platelet samples/ensembles apart. This part of the classification can be improved by comparing the KDE distributions to a larger number of platelet images, as well as by including a larger number of platelets to establish the KDE distributions in the first place. As a way to study how the classification depends on the number of platelet images included, we investigated the variability in the combined classification probabilities of Fig. 6, and how it depended on the number of selection rounds of 6 × 10 platelet images. The classification probabilities, with standard deviations (SDs), are shown in Additional file 1: Fig. S11. As expected, the SDs increase with a decreased number of boot-strapping rounds, as a consequence of the heterogeneity (with multiple biological and technical replicates) within the different pools of platelet images. In spite of this heterogeneity however, the SDs were not more than around 10% on average with 10 rounds, and maximally around 20–30%, comparing classifications based on one single selection round. For 100 or 1000 rounds the SDs of the correct (diagonal) classifications were at most a few percent. While larger sets of platelet images (e.g., allowing a larger number of selection rounds from a larger pool of platelet images) reduce classification variability, it will also open for the use of experimental images in the dictionary learning, as an alternative to the simulated images used in this work. Furthermore, including additional and/or other platelet proteins than the 6 proteins used in this study can likely also lead to additionally improved classification performance. One such protein candidate is p-selectin, for which STED SRM could clearly reveal spatial redistribution patterns in platelets co-cultured with cancer cells, or subject to chemical activations [32]. In the KDE-plots in Fig. 3, clear differences can be seen for some proteins and image parameters between the different co-culturing conditions, while the plots are quite similar for other proteins and image parameters. Another potential improvement possibility can thus be to consider a weighted contribution for the different image parameters in the calculation of the probability scores (p-scores) of the different proteins, as well as for the different proteins in the secondary classification, when calculating the combined probabilities.

Although our classifications are based on protein distribution features within individual platelets, it is ensembles of platelets from different categories we want to distinguish from each other. Given the typical platelet concentrations in blood (~ 400 × 10^9^/l) even minute volumes contain a large number of platelets. We can therefore also label different sets of platelets from the same sample with one or two different proteins, and then expand the number of proteins included in the classification by labeling other proteins on additional sets of platelets, from the same platelet sample/ensemble. From a practical point of view, to enhance the classification strength it is likely easier to increase the number of platelets included than to increase the number of different proteins analyzed, with the concomitant need for separate handling and labeling of multiple sets of platelets in each sample. Nonetheless, the acquisition, analysis and classification procedures established in this work are more automatized and objective, and they are well compatible with further upscaling with further enhanced throughput. This would allow both higher number of platelets and an increased number of different proteins to be accommodated. Moreover, high-throughput image-based analyses are currently under strong development, also for platelet investigations [42]. By implementing concepts from this development into the classification of platelets based on SRM images and nanoscale redistribution of specific proteins, as presented in this study, there is likely room for further improvements.

## Conclusions

Fluorescence-based STED SRM allows us to resolve nanoscale spatial distribution patterns of specific proteins in platelets, at a resolution of a few tens of nanometers. This gives us prerequisites to look into the central role of platelets in cancer progression and metastasis, where altered storage and selective release from platelets of specific tumor-promoting proteins is believed to be a major mechanism. While EM can resolve storage granules and other morphological features of platelets with even sub-nanometer resolution, SRM offers a significantly higher throughput, easier sample preparation, protein-specificity and high sensitivity. Nonetheless, in current practices of SRM the speed of acquisition and analysis is still relatively slow. This represents a major limitation, which makes it difficult to conduct statistically significant analyses on a larger number of platelets.

In this work, we have established a fast, streamlined, and objective acquisition and analysis procedure based on STED SRM. Thereby, we could significantly increase the amount of SRM image data compared to previous SRM studies. With this procedure, we recorded about 100 STED platelet images each, for six different proteins (VAMP7, VAMP8, STX11, SNAP23, TSP1, and VEGF) and for five different categories of platelets; incubated with cancer cells (MCF-7, MDA-MD-231, EFO-21), non-cancer cells (MCF-10A), or no cells at all (R). By structural similarity and protein cluster analyses of the platelet images, and by comparing KDE probability functions of the generated image parameters for the different platelet categories, we could identify nanoscale alterations in the protein distributions, not evident by visual inspection of the images, and allowing us to classify the platelets into their correct categories.

The fast, streamlined and objective acquisition and analysis procedure established in this work makes it possible to overcome a major limitation of SRM-based platelet diagnostics. In our study, we could statistically identify differences in nanoscale protein distribution patterns between the different platelet categories, requiring SRM to be resolved, but too subtle to be clearly detected from a lower number of platelet images. This confirms the role of SNAREs and angiogenesis-regulating proteins in platelet-mediated cancer progression and suggests redistribution of nanoscale protein patterns in platelets as an interesting future basis for cancer diagnostics and treatment monitoring.

## Supplementary Information

**Additional file 1: Figures S1–S11,** with examples of images and FRC analyses, and with classification probability matrices for all combinations of proteins and for different technical and biological replicates, are available online.

## Data Availability

Raw data of this study, including all platelet SRM images, the major settings used in the analyses, and the classification data can be assessed at https://doi.org/10.5281/zenodo.6508428.
