# Supplementary material for: Fast, streamlined fluorescence nanoscopy resolves rearrangements of SNARE and cargo proteins in platelets co-incubated with cancer cells
Source: J Nanobiotechnology. 2022 Jun 21;20:292. doi: 10.1186/s12951-022-01502-w (PMC9210740; doi:10.1186/s12951-022-01502-w)
Supplement: Supplementary file 1 — Additional file 1: Figures S1–S11, with examples of images and FRC analyses, and with classification probability matrices for all combinations of proteins and for different technical and biological replicates, are available online. [file 12951_2022_1502_MOESM1_ESM.docx]

**Supplementary Information:**

**Fast, streamlined fluorescence nanoscopy resolves rearrangements of SNARE and cargo proteins in platelets co-incubated with cancer cells.**

Jan Bergstrand^1,+^, Xinyan Miao^1,+^, Chinmaya Venugopal Srambickal^1,+^, Gert Auer^2^, Jerker Widengren^1,*^

^1^ Royal Institute of Technology (KTH), Department of Applied Physics, Experimental Biomolecular Physics, Albanova Univ Center, SE-106 91 Stockholm, Sweden

^2^ Karolinska Institutet, Department of Oncology-Pathology, K7, Z1:00, Karolinska University Hospital, 171 76 Stockholm, Sweden

^*^ To whom correspondence should be addressed. E-mail: [jwideng@kth.se](mailto:jwideng@kth.se)

^+^ These authors contributed equally

**Fourier Ring Correlation (FRC) analysis:**

FRC analysis was applied to find the effective resolution of the microscope at the imaging conditions and of the deconvolved images used for the analysis and classification as described in the main text. Single image FRC analysis was performed by splitting 400 x 400 pixel STED images (or 200 x 200 pixel confocal images) from an ROI (region of interest) of 4µm x 4µm into two images of 200 x 200 pixels (or 100 x 100 pixels for the confocal images). A gaussian smoothing (Guassian filter in ImageJ Fiji) was performed on the STED images to reduce the photon noise. The maximum spatial frequency in the x axis is dependent on image size and pixel size, and was about 25 µm^-1^ for the STED sub-images. The resolution was calculated by taking the inverse of the spatial frequency at which the FRC crosses the threshold. The procedure for splitting single images for FRC and the 1/7 threshold for resolution calculation was adopted from [1]. In the procedure we considered analyses of single split sub-image pairs to be sufficient, since there was no apparent ellipticity in the Fourier space for the platelet images. We used Python code adopted from [2] for the two image FRC analyses, modified for the dataset presented in the main text.


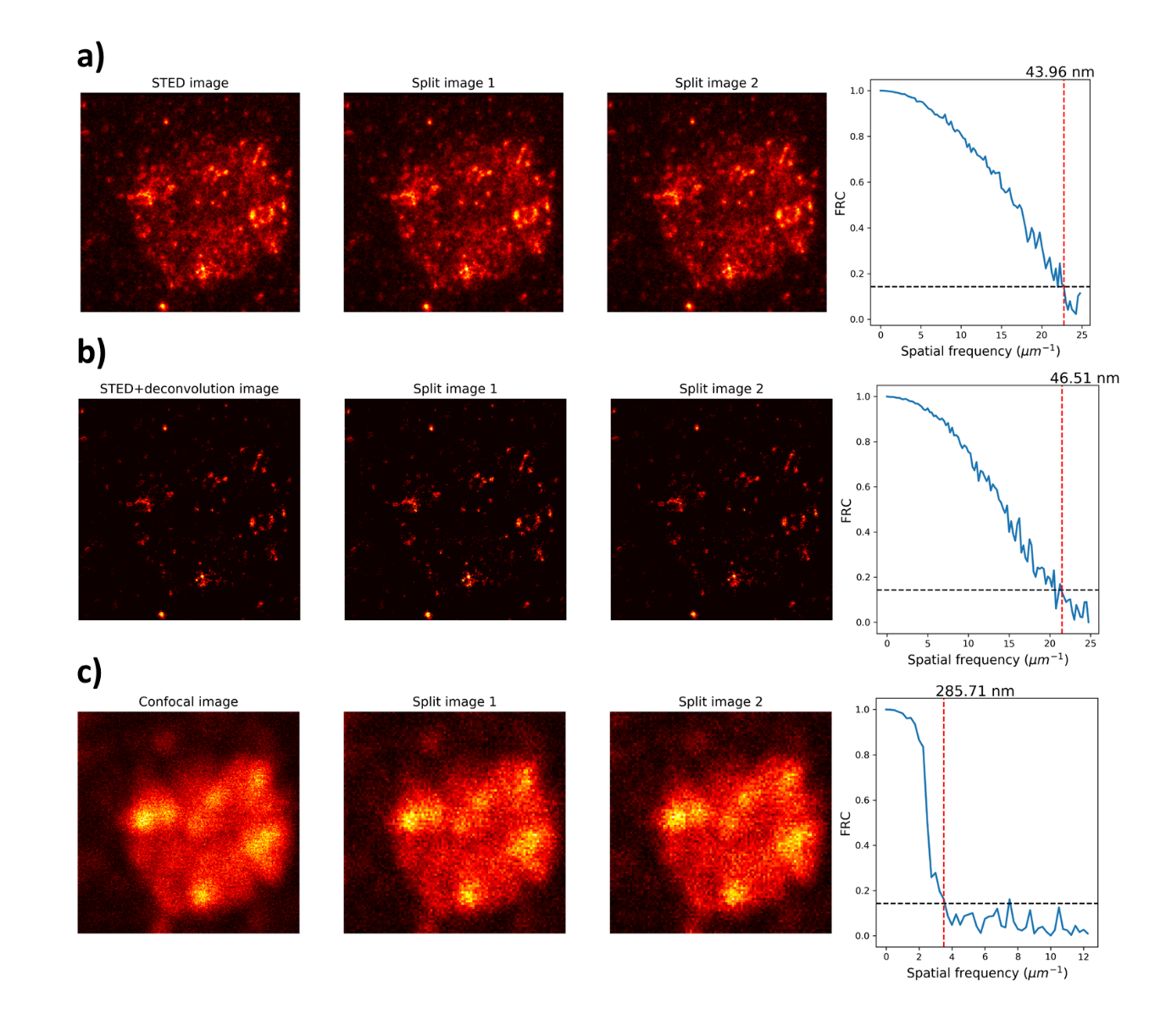
**Figure S1**: Example of an FRC analysis for a platelet image (VAMP7/231). The image was split into two sub images (Split image 1 and 2) to obtain the two images for the FRC analysis. FRC for the a) STED image with an effective resolution of 43.96 nm b) deconvolved STED image with an effective resolution of 46.51nm c) confocal image with an effective resolution of 285.71nm.


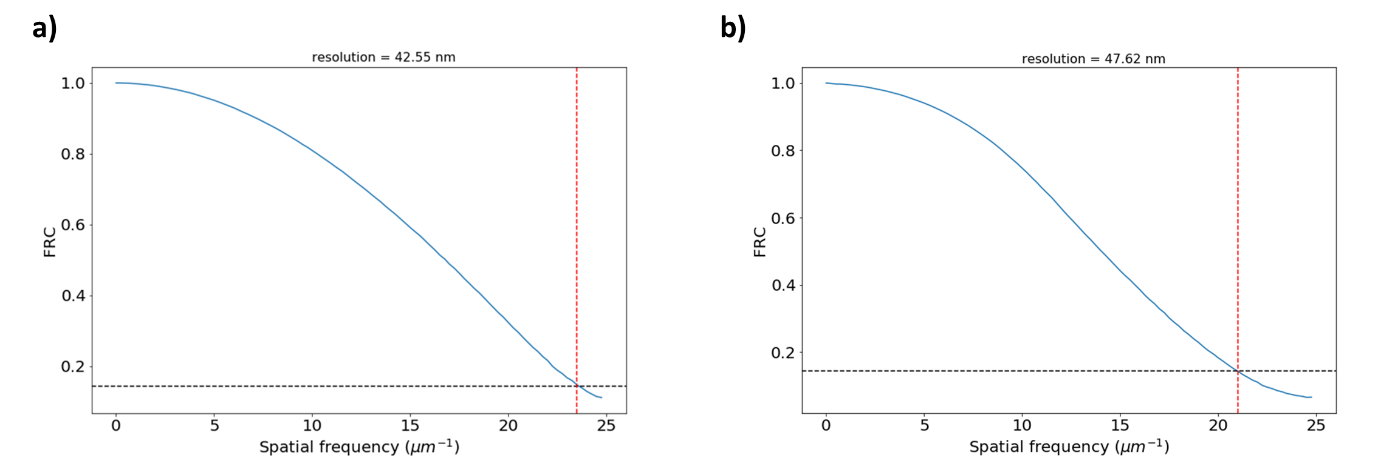


**Figure S2:** Averaged FRC curves for a) all SRM STED images included in the classification, and b) corresponding deconvolved STED images, from all 6 different proteins and the 5 different co-culturing conditions*.*

**Simulated platelet images for dictionary learning:**

The method for simulating platelet images is same as described in the supplementary information of [3]. Briefly, the dictionary was trained on 20000 computer simulated training images (4µm×4µm corresponding to 400x400 pixels), mimicking platelets with a random distribution of proteins. These images were generated using Matlab2013b and contained cluster-like structures of dots (convolved with a Gaussian point spread function), set to vary in size between 20 and 40 nm, and to be randomly and uniformly distributed (between 5 and 500 dots per image) within an elliptic area with minor and major axis randomly distributed between 1-4 µm in size. The brightness of the dots was randomly distributed (such that signal-to-noise ratio took on values between 2 and 20) to resemble images of platelets. Training on such images yields a dictionary that mimicks a collection of platelet images with random distribution of protein clusters.

**Classification matrices for combinations of proteins for secondary classifications:**


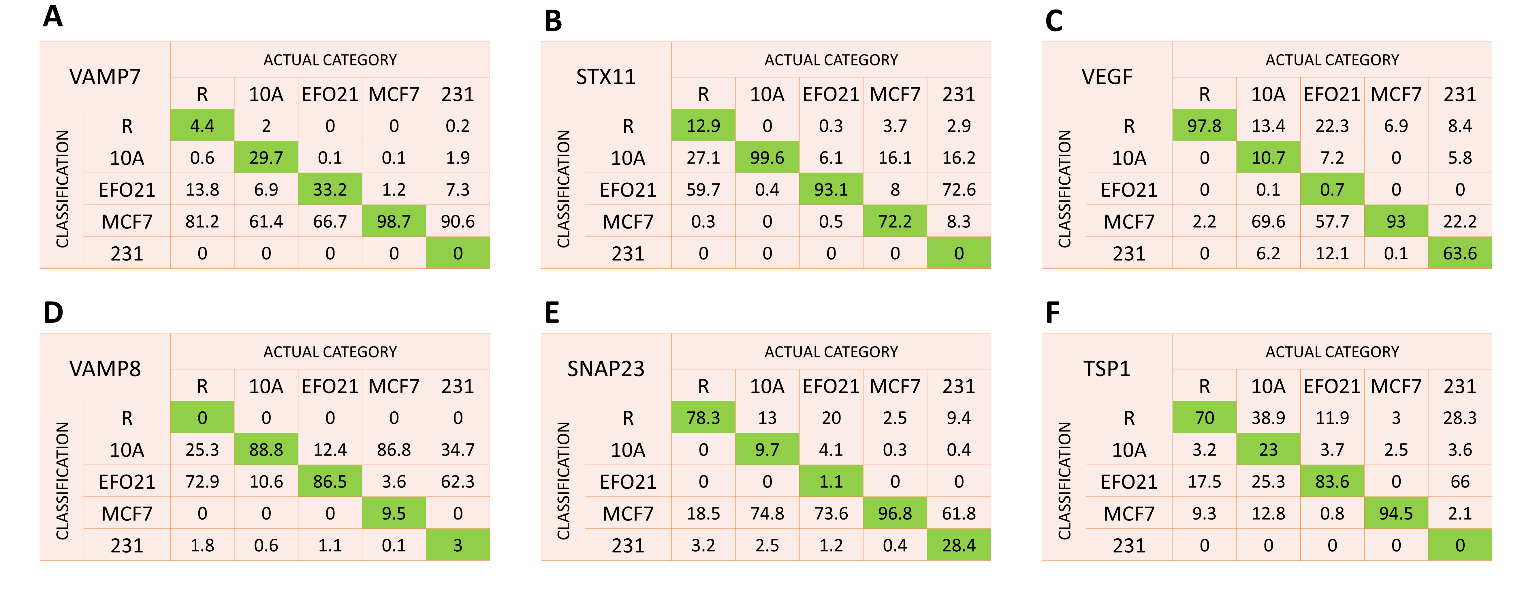


**Figure S3** : Classification matrices generated by using the combined probability ($\Psi_{N}\left( m | \bar{k} \right)$, Eq. 3) method described in the main text, in this case for individual proteins (N = 1) . When a combination of at least 2 proteins (N =2) is used, complementary strengths of the proteins can be utilized for improved classification.


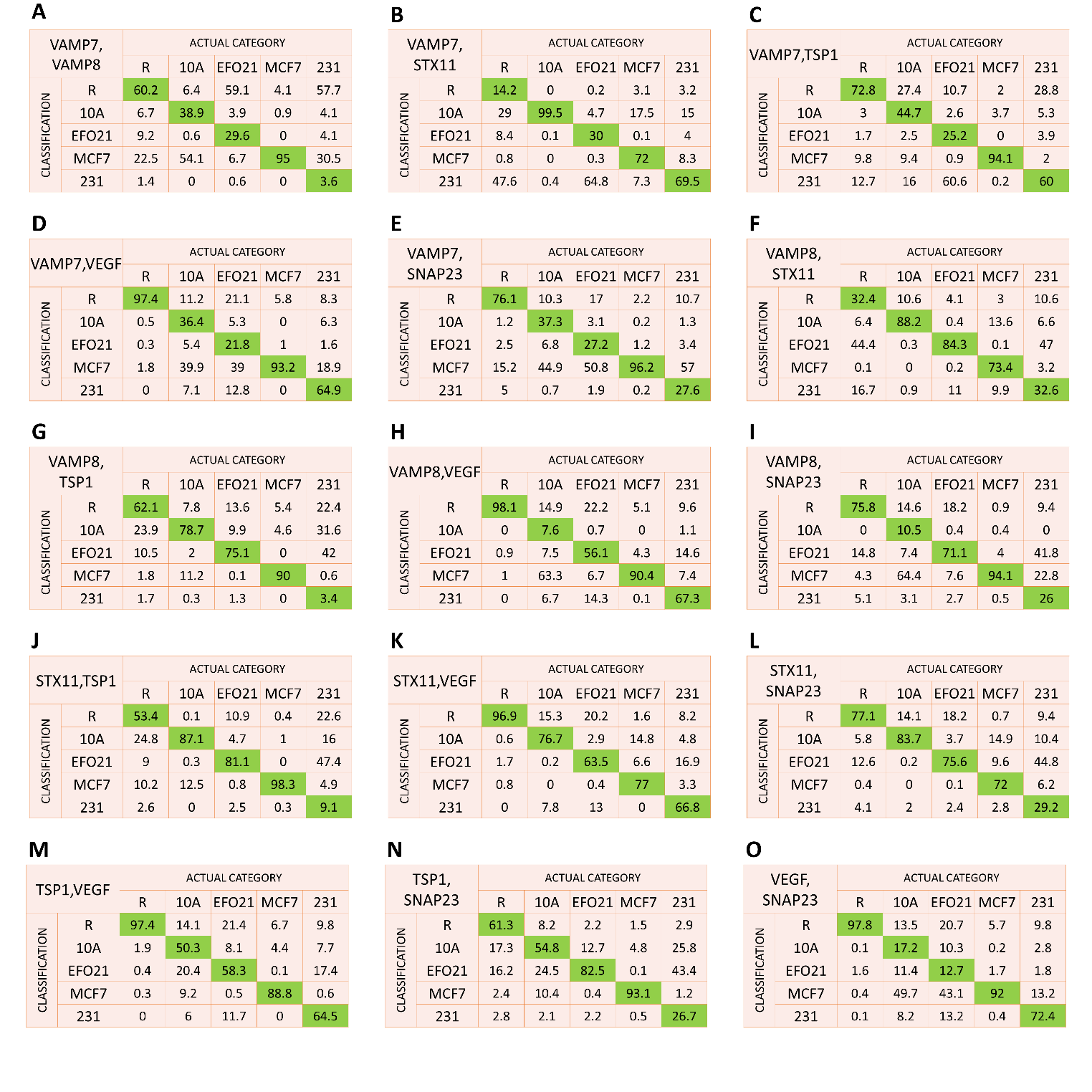


**Figure S4** : Classification matrices generated by combining the complementary classification strengths of combinations of two proteins.


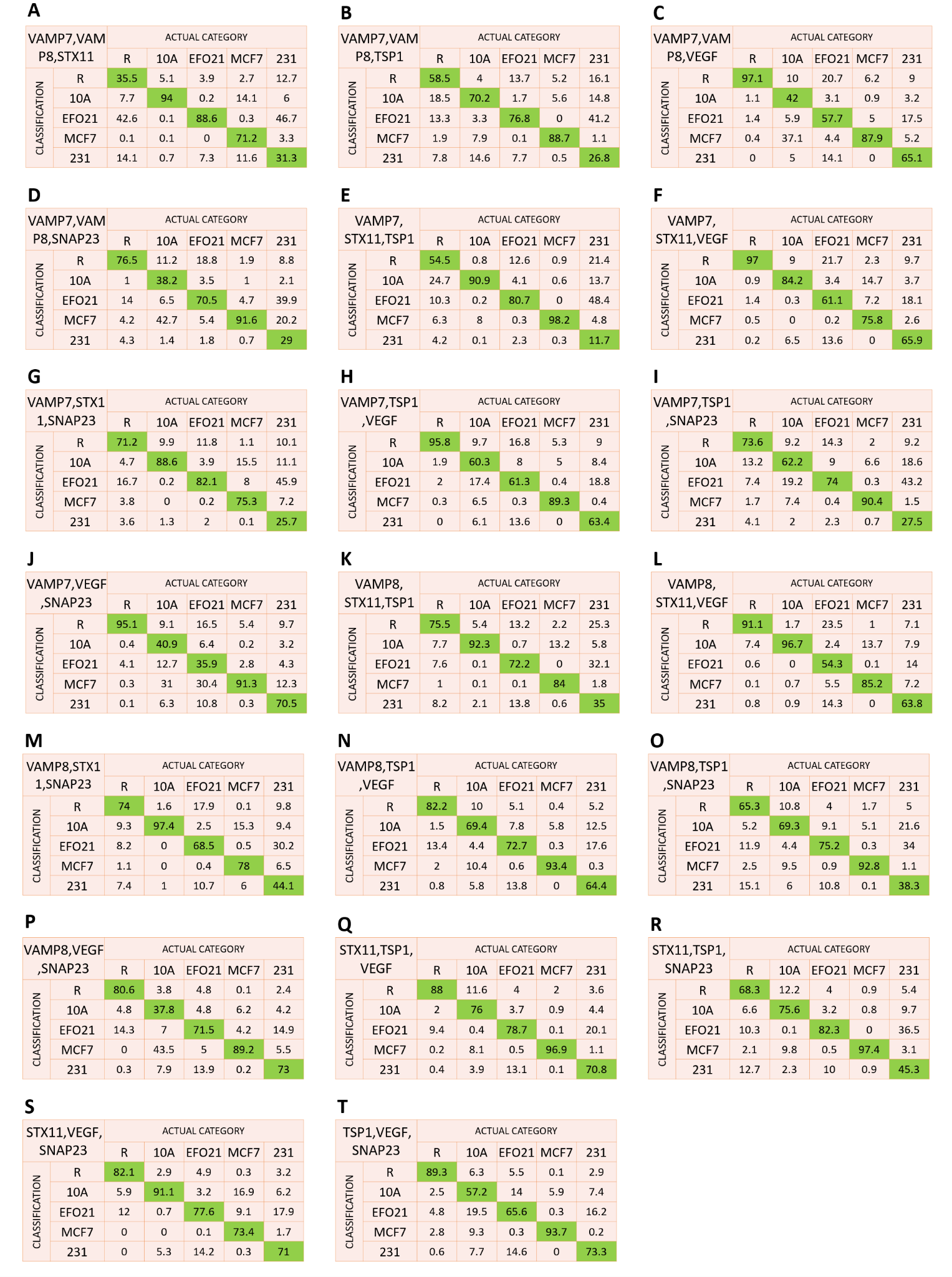


**Figure S5** : Classification matrices generated by combining the complementary classification strengths of combinations of three proteins.


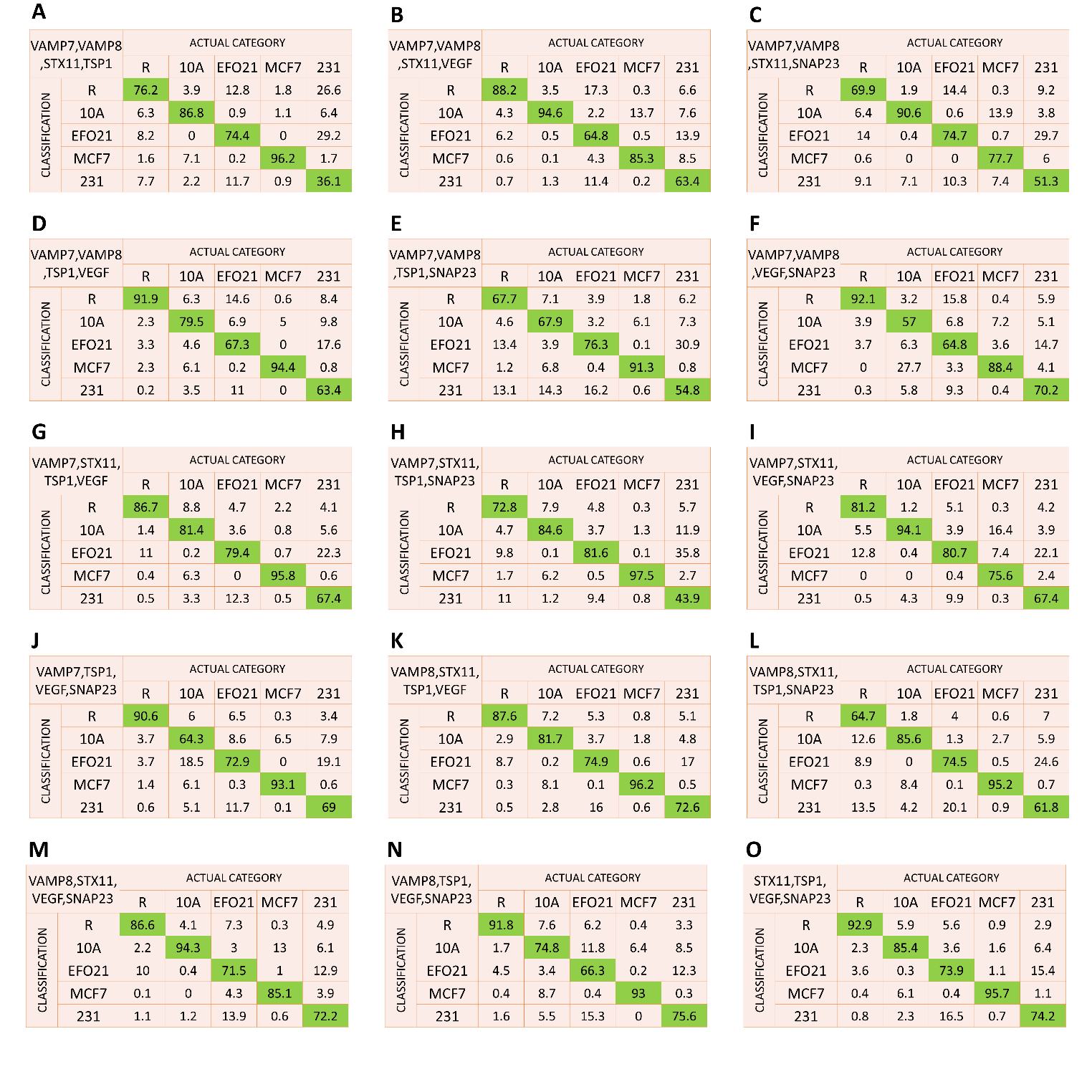


**Figure S6** : Classification matrices generated by combining the complementary classification strengths of combinations of four proteins.


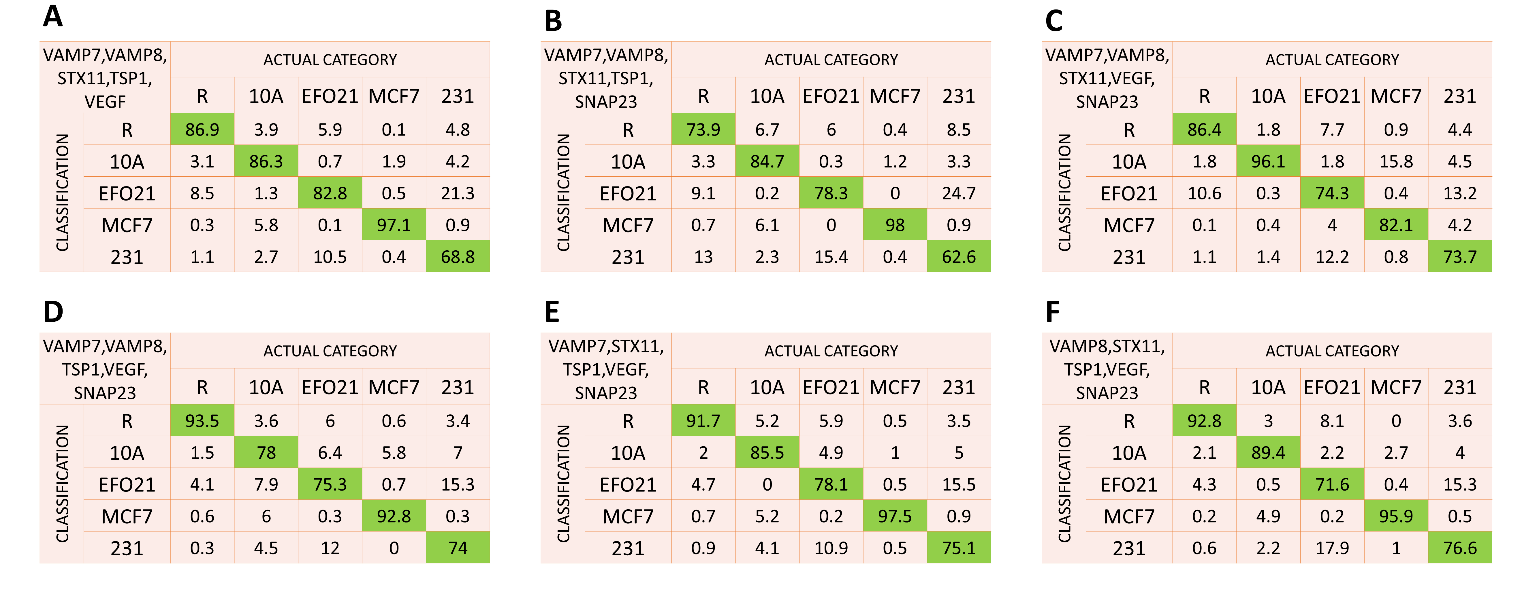


**Figure S7** : Classification matrices generated by combining the complementary classification strengths of combinations of five proteins.


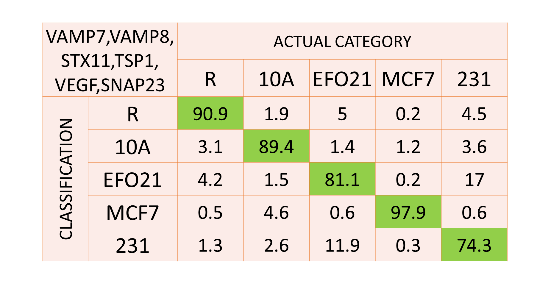


**Figure S8** : Classification matrices generated by combining the complementary classification strengths of combinations of all six proteins.


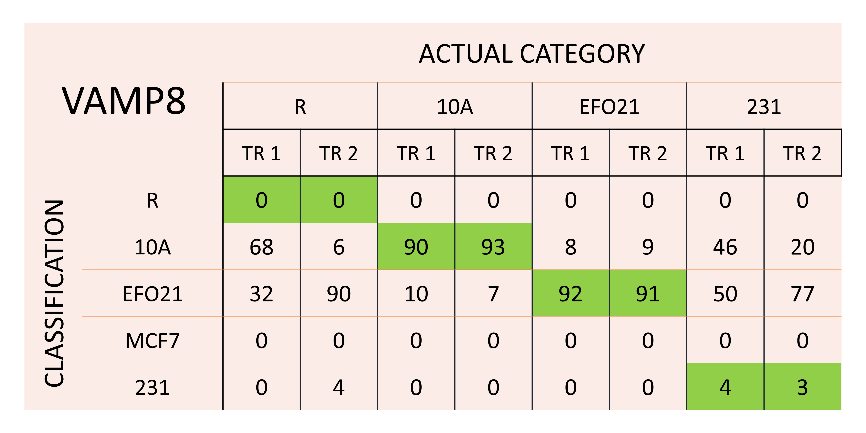


**Figure S9:** Classification probability matrix based on STED SRM images from different technical replicates (TR1 & TR2) of VAMP8-stained platelets within different actual categories. The matrices were generated according to the classification procedure described in the main text, randomly selecting ten platelet images from each of the technical replicate subsets, in this case with the number of selection rounds set to 100 (adapted to the number of platelet images in each subset). TR1 included n=92,81,85,51 and TR2 included n =87,174,56,72 platelets for each of the co-culturing condition (R,10A,EFO21, 231). It can be noted that while the classification in some cases can flip between two outcomes (in the classifications of R and 231), there is otherwise a good reproducibility between the technical replicates, and that the technical replicates (representing only a part of the total platelet images recorded for this protein) also are well in agreement with the total classification outcome of this protein (See VAMP8 matrix in Figure 5). Importantly, for conditions where the classification strength of VAMP8 was found to be strong (10A and EFO21) the reproducibility is high.

**
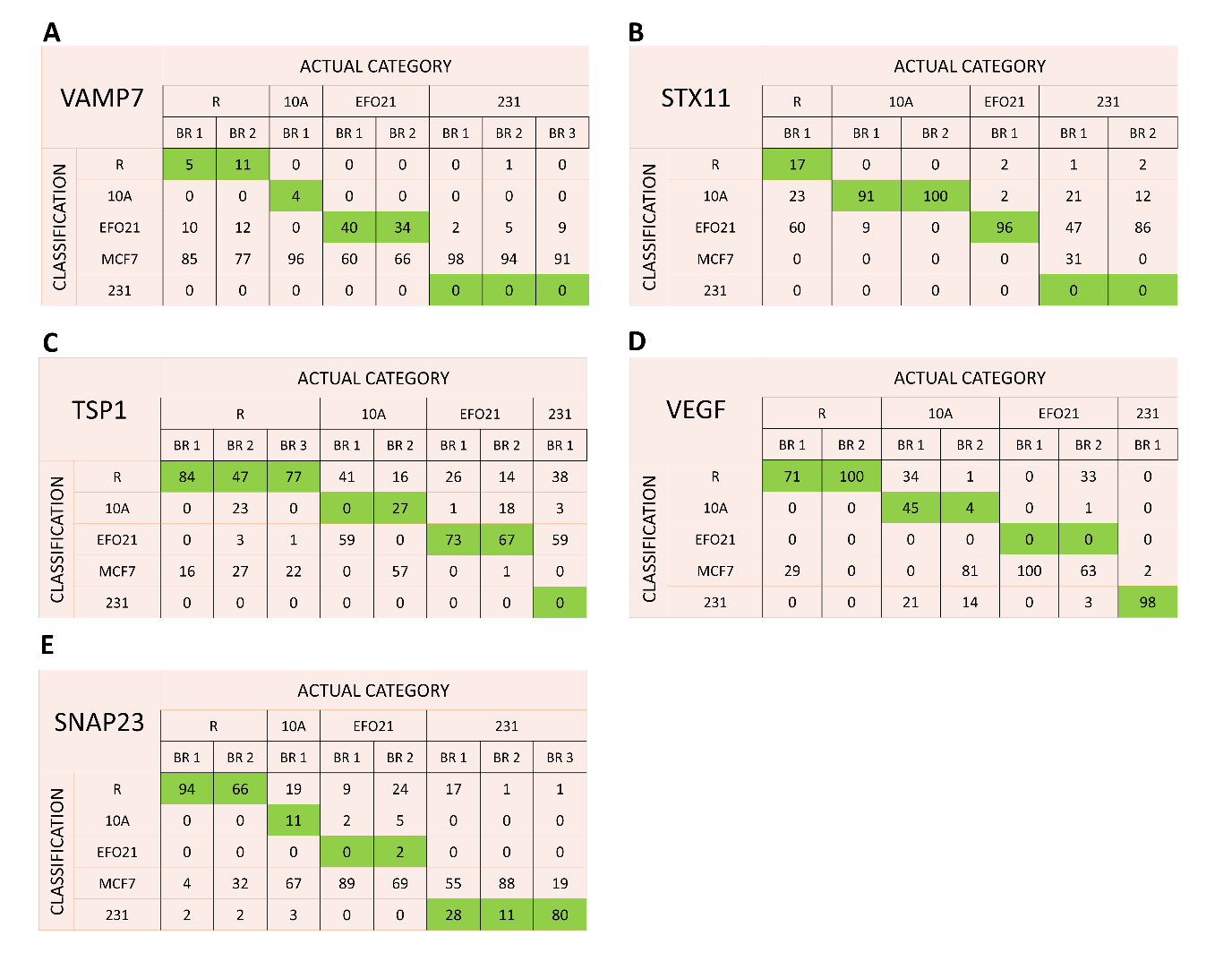
 Figure S10:** Classification probability matrices based on STED SRM images of platelets stained for different individual proteins (except for VAMP8), of different actual categories, and from different biological replicates (BR1, BR2 or BR3). The matrices were generated according to the classification procedure described in the main text, randomly selecting ten platelet images from each of the biological replicate subsets of images (20-140 images per subset), in this case with the number of selection rounds set to 100 (adapted to the number of platelet images in each subset). From the matrices above, we find reasonable reproducibility in the classification, between the identified biological replicates (particularly in the classification of platelets co-cultured with or without cancer cells), and in relation to the total classification outcome, based on the total group of platelets within each co-culturing category (Figure 5). In a few cases (TSP/10A, VEGF/10A, SNAP23/231) there is some disagreement in the classifications (which may also be expected to some degree between biological replicates). However, if instead grouping the classification into platelets co-cultured with cancer cells (EFO21, MCF7, 231) or no cancer cells (R, 10A), there is, with one exception (VEGF/10A), good agreement between the biological replicates, and between the identified biological and the total group of classified platelets (Figure 5).


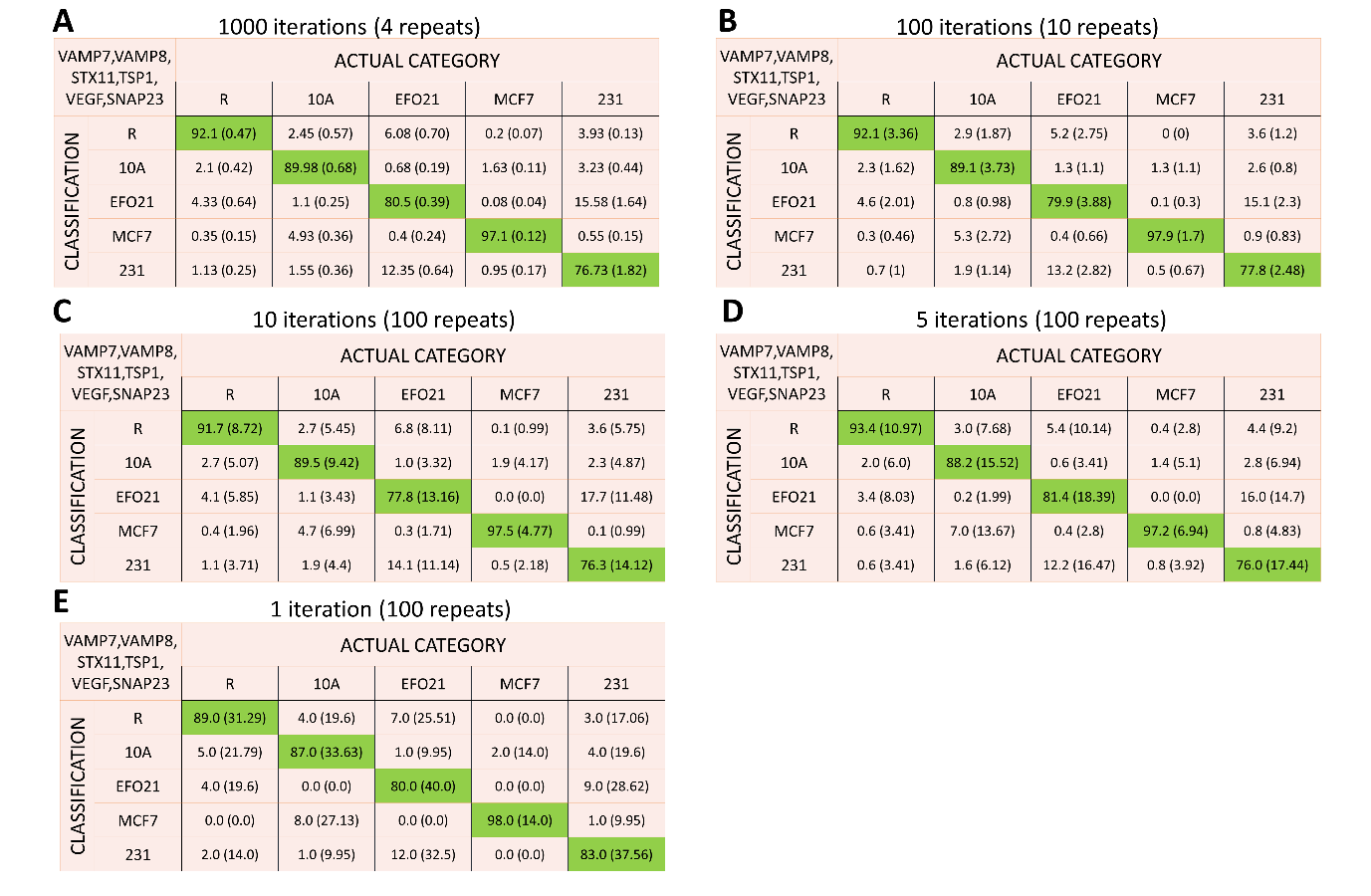


**Figure S11**: Classification matrices, showing means and standard deviations of classification probabilities, and how they vary with the number of iterations used in the classification, i.e. the number of times 6x10 platelet images were selected out of the different specific pools of images (grouped into stained protein and platelet category). A) 1000 iterations (i.e. selction rounds) repeated four times B) 100 iterations (10 repeats) C) 10 iterations (100 repeats) D) 5 iterations (100 repeats) E) 1 iteration (100 repeats). For all matrices, the classifications were based on the same total pool of platelet images, as also used for the classification matrix of Figure 6.

Corresponding classification matrices were also generated for individual protein classifications, and have been added into the uploaded raw data file of this manuscript. They show the same trends as for the 6 protein classifications. Particularly, for the strong classifications of the different proteins, the classification probabilities remained high also with fewer rounds of boot-strapping, and with variabilities comparable to the corresponding ones for the 6 protein classifications.

**References:**

1. Koho S, Tortarolo G, Castello M, Deguchi T, Diaspro A, Vicidomini G. Fourier ring correlation simplifies image restoration in fluorescence microscopy. Nature Communications. 2019 Jul 15;10(1):1-9.
2. <https://github.com/s-sajid-ali/FRC>
3. Bergstrand J, Xu L, Miao XY, Li NL, Oktem O, Franzen B, et al. Super-resolution microscopy can identify specific protein distribution patterns in platelets incubated with cancer cells. Nanoscale. 618 2019;11(20):10023-33
